# FV-MViT: Mobile Vision Transformer for Finger Vein Recognition

**DOI:** 10.3390/s24041331

**Published:** 2024-02-19

**Authors:** Xiongjun Li, Jin Feng, Jilin Cai, Guowen Lin

**Affiliations:** College of Physics and Optoelectronic Engineering, Shenzhen University, Shenzhen 518060, China

**Keywords:** mobile vision transformer, finger vein recognition, light-weight network, center loss

## Abstract

In addressing challenges related to high parameter counts and limited training samples for finger vein recognition, we present the FV-MViT model. It serves as a lightweight deep learning solution, emphasizing high accuracy, portable design, and low latency. The FV-MViT introduces two key components. The Mul-MV2 Block utilizes a dual-path inverted residual connection structure for multi-scale convolutions, extracting additional local features. Simultaneously, the Enhanced MobileViT Block eliminates the large-scale convolution block at the beginning of the original MobileViT Block. It converts the Transformer’s self-attention into separable self-attention with linear complexity, optimizing the back end of the original MobileViT Block with depth-wise separable convolutions. This aims to extract global features and effectively reduce parameter counts and feature extraction times. Additionally, we introduce a soft target center cross-entropy loss function to enhance generalization and increase accuracy. Experimental results indicate that the FV-MViT achieves a recognition accuracy of 99.53% and 100.00% on the Shandong University (SDU) and Universiti Teknologi Malaysia (USM) datasets, with equal error rates of 0.47% and 0.02%, respectively. The model has a parameter count of 5.26 million and exhibits a latency of 10.00 milliseconds from the sample input to the recognition output. Comparison with state-of-the-art (SOTA) methods reveals competitive performance for FV-MViT.

## 1. Introduction

Common biometric identification methods include facial recognition [1], fingerprint recognition [2], and iris recognition [3], all of which have gained widespread adoption. However, it is essential to acknowledge the noteworthy limitations of these methods. For instance, facial recognition accuracy can be negatively affected by factors such as lighting, angles, and obstructions. Fingerprint recognition accuracy decreases in dry conditions or with skin damage. Iris recognition faces limitations in widespread usage due to its high device cost. In contrast, finger vein recognition leverages its inherent advantages to overcome the shortcomings of conventional biometric techniques, offering the following natural advantages: (1). Enhanced security: finger veins are located beneath the skin’s surface and not easily visible or accessible, making them resistant to forgery and attacks [4]. (2). Improved recognition accuracy: finger vein recognition is less affected by external factors such as lighting, angles, and obstructions when compared to conventional techniques. (3). Greater stability: finger vein recognition captures an individual’s internal biological feature, which remains stable and unchanged with age, rendering it less susceptible to external interference [5].

Due to the distinct advantages mentioned above, finger vein recognition has garnered considerable attention from researchers. The technique primarily consists of four stages: image acquisition, image preprocessing, feature extraction, and feature matching. Feature extraction, a critical component, significantly influences the performance and accuracy of the system. Before 2015, finger vein feature extraction relied on traditional image processing and statistical methods, collectively known as traditional algorithms. These methods include Histogram of Oriented Gradients (HOG) [6], Gabor filters [7], Principal Component Analysis (PCA) [8], Local Binary Patterns (LBP) [9], Scale-Invariant Feature Transform (SIFT) [10], Speeded-Up Robust Features (SURF) [11], and Discriminative Multi-Modal Feature Coding (DMFC) [12]. Traditional algorithms often generate features tailored to specific problems and datasets, limiting generality and potentially leading to suboptimal results in diverse scenarios. Moreover, these methods focus on extracting low-level features like edges and textures without the ability to automatically learn and capture higher-level and abstract features, consequently impeding the models’ expressive power. Furthermore, the computational complexity of traditional feature extraction methods is relatively high, rendering them unsuitable for processing large-scale data and potentially adversely affecting real-time performance and overall efficiency.

Deep Convolutional Neural Networks (CNNs) offer several advantages over traditional algorithms when used for finger vein recognition. They facilitate automated feature learning without manual feature design, exhibit robustness, and support transfer learning through fine-tuning for specific finger vein recognition tasks. While CNNs excel at local feature extraction, they face challenges with large parameter sizes, limiting their deployment on mobile devices (e.g., AlexNet [13], GoogleNet [14], and ResNet [15]). On the other hand, Transformer [16] excels at capturing long-range dependencies and extracting global features. Vision Transformer (ViT) [17], the first to apply Transformers to visual tasks, depends on abundant training data, posing challenges for finger vein recognition due to limited datasets. To overcome this challenge, Ze Liu et al. [18] introduced the Swin Transformer, applying a sliding window mechanism to ViT for local information extraction. Both ViT and Swin Transformer are computationally and parameter-wise intensive, leading to the proposal of MobileViT [19] by Sachin Mehta et al. MobileViT combines the strengths of CNNs and Transformers, reducing parameters without compromising accuracy, making Transformer models more suitable for deployment. However, applying MobileViT to finger vein recognition faces two key challenges: the limited samples may cause overfitting, and the reduction of parameter counts may decrease accuracy. To address these challenges, this paper proposes the FV-MViT algorithm, a lightweight algorithm for finger vein recognition combining CNNs and Transformer models, introducing a soft target center cross-entropy loss function. FV-MViT includes the Mul-MV2 Block and the Enhanced MobileViT Block. The Mul-MV2 Block first performs multi-scale convolution and dual-channel stacking and then utilizes point convolution to fuse the two streams of local features, thus enhancing the model’s recognition accuracy. Enhanced MobileViT Block removes the large-scale convolution before MobileViT Block FOLD, transforms the self-attention in Transformer into separable self-attention with linear complexity, and optimizes the convolution after UNFOLD with depthwise separable convolution, aiming to extract global features and reduce parameter counts.

The primary contributions of this paper are as follows:

1. A lightweight finger vein recognition network, FV-MViT, based on CNN and Transformer, is proposed. It achieves competitive results when compared to the state-of-the-art (SOTA) in terms of parameter count, latency, and recognition accuracy.
2. During the training of deep models, a combined loss function of soft target cross-entropy and center loss is employed. This loss function improves the model’s generalization, reduces intra-class distances, and increases inter-class distances, thereby enhancing the model’s recognition accuracy.
3. We introduce a method for image preprocessing that utilizes adaptive histogram equalization with contrast limiting to enhance images. We locate the mini-regions of interest (Mini-ROI) area by using the coordinates corresponding to the peak value after the accumulation of grayscale values of row and column pixels. This algorithm effectively extracts the relevant region of finger veins, reducing the impact of redundant information on the accuracy of recognition.

The rest of this paper is organized as follows: Section 2 provides an overview of related work in finger vein recognition. Section 3 elaborates on the proposed algorithms. Section 4 presents experimental results to demonstrate the performance of the proposed algorithms. Section 5 concludes the paper.

## 2. Related Work

### 2.1. Finger Vein Image Preprocessing Method

**Figure 1:**
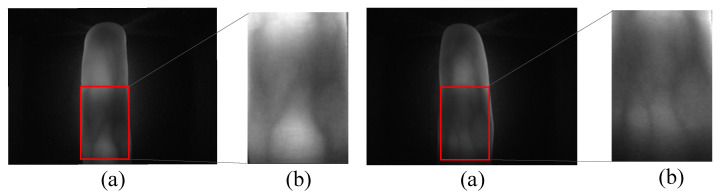
Feature extraction of Mini-ROI in the USM Dataset, (**a**) original image and (**b**) image processed with feature extraction of Mini-ROI.

Effective preprocessing is essential for algorithms employing deep learning in feature extraction and matching, particularly in the context of finger vein images. The extraction of Mini-ROI proves highly beneficial for the recognition of finger vein images by eliminating redundant information, thus improving recognition accuracy, as demonstrated by the localization result from the USM dataset, illustrated in Figure 1. Fang Yuxun et al. [20] introduced three methods for extracting Mini-ROI: Entropy with Displacement Constraints (ENTR), Correlation with Displacement Constraints (CORR), and Center (CENT). These methods aim to mitigate the impact of displacement by simultaneously reducing intra-class distances while preserving inter-class differences. Yang Lu et al. [21] leveraged the structural characteristics of finger veins and the principles of optical system design to precisely localize the ROI for finger vein recognition. Yang Jinfeng et al. [22,23] employed the distal interphalangeal joint as the reference point for localization and combined a sub-window scheme and pixel value accumulation to accurately determine the position of the vein’s ROI. Additionally, they normalize ROI images to mitigate shape differences.

### 2.2. Lightweight Finger Vein Recognition Algorithm Based on Deep Learning

Given the portability requirement for finger vein recognition on mobile devices, the development of lightweight neural networks for finger vein recognition is an important research focus. Recent research in lightweight neural networks has primarily focused on convolutional neural networks. This includes the use of depth-wise separable convolutions in MobileNet [24], the introduction of inverted residual connections and linear bottlenecks in MobileNetV2 [25], and the optimization of network structure and parameters using Neural Architecture Search (NAS) and NetAdapt in MobileNetV3 [26]. These advancements aim to achieve optimal performance and efficiency on various hardware platforms. The majority of studies on lightweight neural networks for finger vein recognition have concentrated on the utilization of convolutional neural networks (CNNs). Shen Jiaquan et al. [27] designed a lightweight finger vein recognition network employing channel stacking, consisting of two modules: the Stem Block and the Stage Block. The Stem Block rapidly convolves input image information, whereas the Stage Block conducts meticulous stacking operations on the extracted features. Employing point convolutions and a three-channel feature stacking method, this network not only enhances recognition performance but also diminishes computational costs effectively. Wang Kaixuan et al. [28] employed a lightweight network design strategy, by integrating multiple receptive field bilinear convolutional neural networks and the Dimension Interaction Attention Mechanism (DIAM) to create a finger vein recognition network. The streamlined design strategy significantly reduces model parameters and computational complexity. This efficiency is further augmented by DIAM, which strengthens interdependencies among channels and spatial aspects, consequently elevating the accuracy of network predictions and classifications. Song Yizhuo et al. [29] proposed an Explicit and Implicit Feature Fusion Network (EIFNet) for finger vein verification, which achieved more comprehensive and distinctive feature extraction by fusing features from binary vein masks and grayscale original images. The authors designed a feature fusion module acting as a bridge between the mask feature extraction module and the contextual feature extraction module to achieve the optimal fusion of features. Junduan Huang et al. [30] proposed an attention mechanism, namely, the joint attention (JA) module, which enables dynamic adjustment and information aggregation in the spatial and channel dimensions of feature maps to focus on fine-grained details. Yiwei Huang et al. [31] introduced an axially enhanced local attention network (ALANet) that used the distribution of vascular topology to infer attention in their study. These researchers achieved high recognition rates using CNN as the network for finger vein feature extraction. However, the challenge in applying CNN lies in the large parameter count, making it impractical for deployment on mobile devices.

In recent years, driven by advancements in computer vision, researchers have begun leveraging Transformers for finger vein recognition. Huang Junduan et al. [32] proposed a novel model, the FV Transformer (FVT), for Finger vein authentication. The FVT consists of four key modules: (1) the conditional position embedding; (2) the weight-shared expanded multilayer perceptron (EMLP); (3) the local information-enhanced feedforward network (FFN); and (4) the expansion-less mechanism (ELM). Qin Huafeng et al. [33] implemented a local attention Transformer framework, which introduced an adjacency matrix to enable local self-attention between adjacent image blocks and the entire view image. This network consists of two stages of transformers for feature extraction and representation. It also utilizes Conditional Position Encodings (CPE) to dynamically generate positional information. Raul Garcia-Martin et al. [34] used Vision Transformers (ViTs) for vein biometric recognition. The authors utilized pre-trained and fine-tuned ViTs to extract unique image features in four variants of Vascular Biometric Recognition (VBR) and conduct vein identification tasks on 14 vascular datasets, achieving excellent results. These researchers have made significant progress using Transformers. Despite their potential, Transformers often face challenges due to their large parameter counts and computationally intensive nature, making it difficult to deploy them on mobile devices. Hence, confronted with the aforementioned challenges in the application of CNN and Transformer to finger vein recognition, there is a pressing need for further research. The integration of CNN and Transformer is pivotal for attaining elevated recognition rates, streamlined design, and minimal latency.

### 2.3. Loss Functions for Finger Vein Recognition

The loss functions play a crucial role in determining the generalization and recognition accuracy of models, particularly for datasets with limited samples of the same class, such as finger vein data. Previously, researchers employed different loss functions for finger vein recognition models. Shen Jiaquan et al. [27] used a triplet loss function to train deep learning models, effectively addressing the issue of sample scarcity in finger vein recognition. Zhao Dongdong et al. [35], by combining the SoftMax with center loss functions, successfully reduced intra-class distances and increased inter-class distances, ultimately improving finger vein recognition. Hou Borui et al. [36] introduced a novel loss function called the arccosine center loss, which combined the SoftMax loss and the arccosine center loss. This approach empowers the network model to capture both inter-class and intra-class information, thereby enhancing recognition accuracy. Each of these loss functions possesses distinct advantages, offering valuable insights for training FV-MViT and addressing the challenges associated with limited training samples.

## 3. Proposed Method

This paper introduces three algorithms meticulously crafted for key stages in the finger vein recognition process: (1) feature extraction of the finger vein Mini-ROI region; (2) a lightweight finger vein recognition and matching algorithm based on CNN and Transformer; and (3) the utilization of a soft target center cross-entropy loss function. The first step effectively removes redundant information from the images, thus significantly reducing noise and data processing requirements. The lightweight finger vein recognition and matching algorithm, utilizing CNN and Transformers, enables efficient training and matching of finger vein pattern images. It utilizes the Adam optimizer and implements dynamic learning rate adjustment using the cosine annealing algorithm to improve neural network recognition accuracy. Finally, we employ the soft target center cross-entropy loss function, which combines soft target cross-entropy loss and center loss. This fusion loss function enhances the model’s generalization ability, increases inter-class distances, and reduces intra-class distances.

### 3.1. Feature Extraction of Finger Vein Mini-ROI

Original finger vein images often contain a fair amount of redundant information, contributing to increased model network complexity, unnecessary resource consumption, and a detrimental impact on recognition algorithm accuracy. Therefore, it is essential to extract the effective region of the finger vein image. The specific algorithm implemented in this paper is referred to as the Mini-ROI feature extraction algorithm. The detailed steps are as follows.

#### 3.1.1. Contrast-Limited Adaptive Histogram Equalization (CLAHE)

The original vein image presents several challenges, including low contrast that impedes effective region identification, obscured local detail features, and substantial interfering noise with feature extraction. Addressing the issue of low contrast involves the application of histogram equalization for contrast enhancement. However, caution is required, as it may lead to the loss of local details and the generation of additional noise due to global contrast enhancement. To better capture local information, a strategy involves dividing the image into blocks, and applying individual histogram equalization to each block. This approach mitigates the risk of losing local information associated with global histogram equalization. By adjusting the contrast of individual image blocks within a defined threshold, noise suppression can be achieved. In combination, these solutions form the Contrast-Limited Adaptive Histogram Equalization (CLAHE) algorithm [37], which contains the following steps: image segmentation for per segmented histogram acquisition, contrast limitation, mapping of blocks based on segmented histograms, and finally, edge elimination through interpolation to remove the block artifacts.

**Figure 2:**
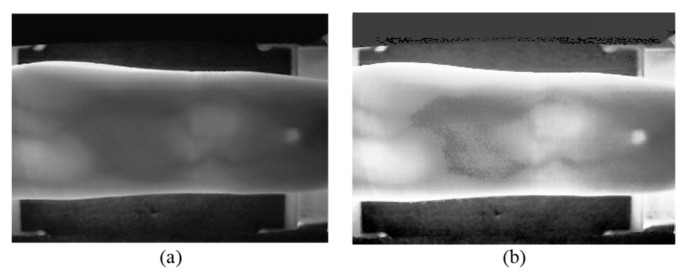
Sampling and preprocessing results, (**a**) original sampled image and (**b**) image processed with CLAHE.

The sampled original image is shown in Figure 2a, while the image processed with CLAHE is shown in Figure 2b. A noticeable difference emerges upon comparison: the local contrast of the processed image is significantly enhanced, the detail features are enhanced, and the noise is also suppressed to some extent.

#### 3.1.2. Mini-ROI Extraction

**Figure 3:**
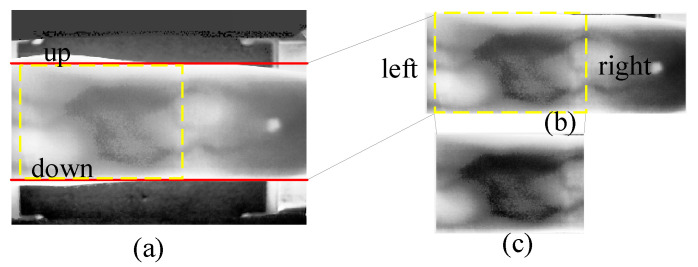
Cropping results. (**a**) Equalized image; (**b**) first Cropping; (**c**) second Cropping. In the figure, the red line represents the upper and lower boundaries, and the yellow line represents Mini-ROI.

**Figure 4:**
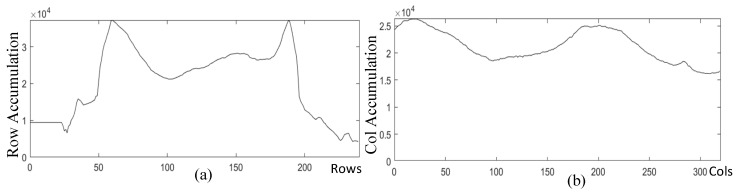
Pixel accumulation images. (**a**) Pixel accumulation image in the row direction; (**b**) pixel accumulation image in the column direction.

Figure 3a displays the result of CLAHE, revealing quite high brightness at the upper and lower finger edges in the equalized image. As brightness increases, so do pixel grayscale values, contributing to higher cumulative pixel grayscale values in the row direction. This characteristic aligns with the essential edge regions that the design intends to identify. Figure 4a exhibits the cumulative pixel grayscale values in the row direction, where two distinct peaks are clearly visible. The left peak corresponds to the horizontal coordinate of the ‘up’ edge in Figure 3a, and the right peak corresponds to the horizontal coordinate of the ‘down’ edge in Figure 3a. Cropping is conducted from the equalized image in Figure 3a to Figure 3b according to the ‘up’ and ‘down’ coordinates.

**Figure 5:**
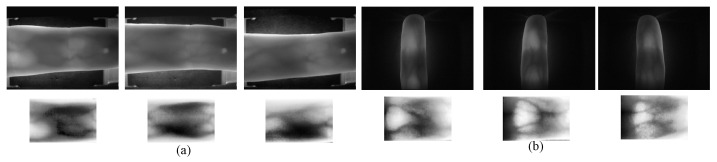
Examples of extracted Mini-ROI, depicting the upper column representing the original finger vein image and the lower representing the extracted Mini-ROI of (**a**) SDU and (**b**) USM.

The goal of Mini-ROI extraction is to obtain the most information-rich area in the vein image. As depicted in Figure 3b, the middle joint of the finger contains the most information, while the fingertip contains the least. Therefore, we should obtain an image of the middle joint of the finger. Figure 4b is the result of pixel grayscale value accumulation in the column direction in Figure 3b. Two peaks in Figure 4b are clearly observed, where the left peak corresponds to the horizontal coordinate of the ‘left’ edge in Figure 3b, and the right peak corresponds to the horizontal coordinate of the ‘right’ edge in Figure 3b. Cropping Figure 3b based on the left and right coordinates yields Figure 3c. Figure 5 illustrates examples of Mini-ROI extracted from the two datasets.

### 3.2. Lightweight Vein Recognition Neural Network FV-MViT Based on CNN and Transformers

**Figure 6:**
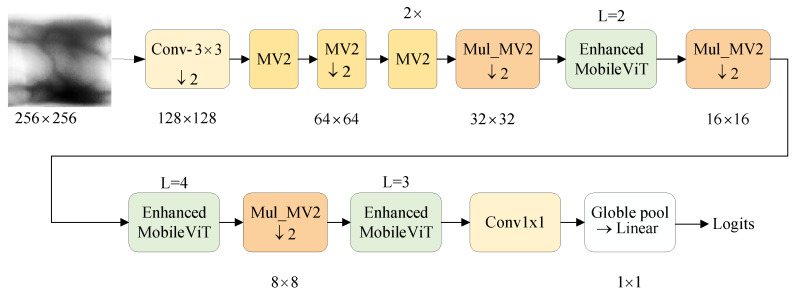
Finger vein feature extraction network.

The design of the FV-MViT network for lightweight finger vein recognition is primarily inspired by MobileViTV1 [19], MobileViTV2 [38], GoogleNet [14], MobileNetV1 [24], and MobileNetV2 [25]. The central concept behind the FV-MViT network design is to eliminate the large-scale convolutions for local feature extraction from the front end of the MobileViT Block. Instead, employing the Mul_MV2 Block conducts dual-path convolutions at different scales while conducting the fusion of local features, thereby accomplishing the extraction of local features. As shown in Figure 6, the Mul_MV2 Block conducts local feature extraction and downsampling, while the Enhanced MobileViT Block performs global feature extraction. The combination of both blocks enables effective feature extraction from finger vein images.

#### 3.2.1. Overall Architecture

The finger vein feature extraction network is shown in Figure 6, with the input finger vein image size at 1 × 256 × 256. Firstly, a 3 × 3 convolution is employed for one-time downsampling to adjust the image size to 16 × 128 × 128. Then, the feature extraction is performed by using four MV2 Blocks, and the image size is adjusted to 64 × 64 × 64. The Mul_MV2 Block then follows, performing three essential functions: multi-scale local feature fusion, down-sampling, and increasing the number of channels in the image. The output image size of the Mul_MV2 Block is 96 × 32 × 32. Following the Mul_MV2 Block is the Enhanced MobileViT Block, responsible for global feature extraction. This design combines the advantages of CNN and Transformer, effectively addressing the issue of the low recognition rate of ViT on small datasets. The subsequent steps involve two identical operations, resulting in an image size of 160 × 8 × 8. As the two datasets adopted have 636 and 492 classes, more channels are needed to improve image classification. A point convolution is used to increase the image’s channel dimension to 640, and the image size is adjusted to 640 × 8 × 8, global pooling is then applied, resulting in 640 × 1. Finally, a fully connected layer is used to output features of either 636 or 492 dimensions.

#### 3.2.2. MV2 Block and Mul_MV2 Block

**Figure 7:**
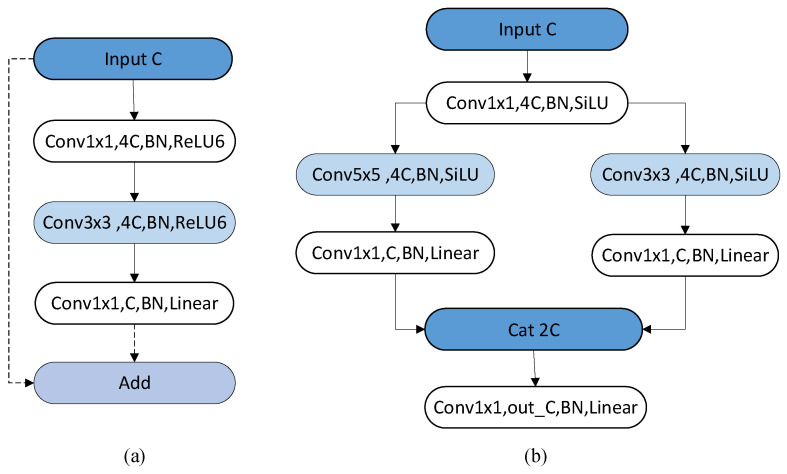
(**a**) MV2 Block; (**b**) Mul-MV2 Block. In the figure, we use the same color for similar structures.

MV2 Block: The MV2 Block, depicted in Figure 7a, initially utilizes point-wise convolution to increase the dimensionality from input dimension *C* to *4C*. The rationale behind this approach is to expand the dimensionality, providing more channels for convolution, which in turn enables the extraction of additional features, thereby enhancing the model’s performance. After the point-wise convolution, batch normalization and ReLU6 are applied. During training, batch normalization is used to normalize parameters for each layer, enabling dynamic adjustments of learning rates, thereby accelerating the convergence speed of the training model. The reason for using ReLU6 is that the data float16 has low precision when running on the mobile terminal, and the nonlinear activation function ReLU6 can effectively avoid the loss of precision. Subsequently, a point convolution is employed to restore the dimensionality back to its original size, facilitating the stacking of information thereafter. After the point-wise convolution, batch normalization is applied, followed by a linear connection. The reason for using a linear connection instead of ReLU is that applying ReLU after mapping from high dimensionality to low dimensionality and then returning to high dimensionality leads to a loss of information. This phenomenon is referred to as a “linear bottleneck” in MobileNetV2. To overcome this linear bottleneck, a linear connection is used instead of ReLU. As shown in Figure 7a, the dashed lines represent residual connections. When the MV2 Block performs downsampling, causing a change in the image scale, residual connections are not employed. When MV2 Block does not perform down-sampling, maintaining the image scale, a residual connection is employed.

Mul-MV2 Block: The Mul_MV2 Block, depicted in Figure 7b, maintains identical overall structures in both pathways. Every pathway incorporates an inverted residual block, employing pointwise convolution to expand dimensionality, followed by convolutions at different scales, and concluding with pointwise convolution to reduce dimensionality. Drawing inspiration from the Inception structure [14], which aims to construct a sparse network architecture that transforms the original high-dimensional image into four low-dimensional images using point convolution. Each path performs convolutions with convolutional kernels of varying scales and the low-dimensional information from each path is then stacked to generate dense data, enhancing the ability to extract diverse local information and optimizing the utilization of computational resources. In line with the multi-scale sampling approach of the Inception structure, this paper increases the dimension of the input data and divides it into two paths for feature extraction. Both paths employ inverted residual connection structures, with the first path employing a 5 × 5 convolution and the second path utilizing a 3 × 3 convolution. Following the output of the data from both paths, stacking is performed using the concat function. Subsequentially, a dimension transformation occurs using point convolution to adjust the dimension to *out_C*. The choice of employing multi-scale convolution in this study stems from the limited quantity of vein samples available for network training. Multi-scale convolution enhances the capability to effectively extract local features from the constrained sample set. Additionally, this paper incorporates the *SiLU* activation function to effectively mitigate the issue of gradient disappearance. As depicted in Equation (1), as *x* approaches 0, the function demonstrates good continuity and differentiability in this region, ensuring smooth variation without abrupt changes. When *x* exceeds 0, the *SiLU* function behaves similarly to the ReLU function, implying linearity in this region.

(1) SiLU(x)=x·sigmoid(x)

#### 3.2.3. Enhanced MobileViT Block

**Figure 8:**
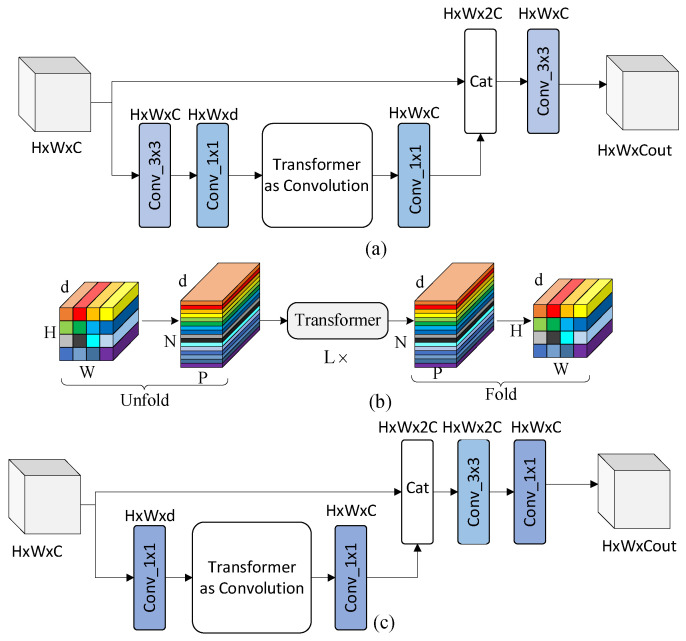
(**a**) Original MobileViT Block structure; (**b**) Transformer as Convolution Block structure; (**c**) Enhanced MobileViT Block structure. In the figure, the first dimension *H* represents height, the second dimension *W* represents width, and the third dimension *d*/*C* represents depth. We use the same color for similar structures.

The structure of the original MobileViT Block is shown in Figure 8a. It takes the input data with a height of *H*, a width of *W*, and a channel number of *C.* The input data undergoes a 3 × 3 convolution to capture local image features. Following this, a point convolution is used for channel transformation, reducing the number of channels to *d*. Next, it is fed into the Transformer as a Convolution Block, where global image features are obtained. The dimensions of the output image features remain unchanged. Subsequently, a point convolution is applied to transform the number of channels back to *C.* The result is then stacked with the original image, resulting in image features with *2C* channels. Finally, the stacked features are fused by using a 3 × 3 convolution, simultaneously adjusting the number of channels, ultimately yielding the desired output with a channel count of *Cout*. The structure of the Transformer as a Convolution Block is shown in Figure 8b, which preserves the positional information of the image through unfolding and folding operations.

The Enhanced MobileViT Block, as shown in Figure 8c, deletes the 3 × 3 convolution because the preceding Mul-MV2 Block has already performed local feature extraction and fusion. Furthermore, in this block, the self-attention mechanism in Transformer is modified to a separable self-attention mechanism with linear complexity [38], reducing the complexity from $O\left(k^{2}\right)$ to $O\left(k\right)$. This transformation results in a reduction of model parameters and a decrease in latency from the sample input to the recognition result output. Channel stacking is performed using the concat operation, resulting in a channel count of *2C*. In this section, we employ depth-wise separable convolutions to improve the convolution in the backend. Initially, the stacked features are fused by using a 3 × 3 convolution, and the output dimension remains *2C*. Subsequently, a point convolution is applied to transform the number of channels into the output dimension. This optimization effectively reduces the number of parameters and enhances the model performance.

### 3.3. Training Algorithm

#### 3.3.1. Data Augmentation

In this paper, Mixup is used for data augmentation [39]. Mixup primarily combines the features of two samples linearly and also combines their corresponding labels linearly to generate new samples and labels. Suppose there are two input samples, $x_{i}$ and $x_{j}$, along with their corresponding labels, $y_{i}$ and $y_{j}$. Mixup generates new samples and labels using the following formulae:

(2) $$x_{k}=\lambda x_{i}+(1-\lambda )x_{j}$$

(3) $$y_{k}=\lambda y_{i}+(1-\lambda )y_{j}$$

Here, $x_{i}$ and $x_{j}$ represent input feature vectors of dimension n, while the target labels $y_{i}$ and $y_{j}$ are one-hot vectors with dimension n. λ is a random parameter from the Beta distribution that is used to determine the weights of the two samples. After mixing the two samples, the input feature vectors are transformed into a vector $x_{k}$ with a certain degree of blending, and the output of the target labels becomes an n-dimensional probability distribution $y_{k}$. This blending operation helps improve the model’s generalization ability and enhances its classification capability for different classes of samples. Furthermore, the obtained n-dimensional probability distribution, $y_{k}$, can be easily input into the subsequent soft-target cross-entropy loss function for computation.

#### 3.3.2. Soft Target Center Cross-Entropy Loss Function

Throughout the model training process, two issues require attention. Firstly, a notable concern arises when the training accuracy significantly surpasses the testing accuracy, indicating a need to improve the model’s generalization ability. In this study, we utilize soft target cross-entropy to address this issue. Compared to traditional cross-entropy loss, soft target cross-entropy offers a smoother optimization space, resulting in a more stable and efficient training process. By representing target labels as probability distributions instead of one-hot vectors, the model avoids excessive confidence in its predictions, ensuring more robust and generalizable training results. Secondly, a challenge arises due to the limited number of available training samples. To tackle the small sample issue, employing center loss [40] can guide feature vectors towards the centers of their respective correct classes. This approach enhances feature representation on limited samples by reducing intra-class variance and enlarging inter-class variance, consequently enhancing classification performance. Thus, the proposed method in this paper combines the soft target cross-entropy loss as the main loss function with a certain proportion of the center loss function during the neural network’s training process. This unified loss function not only enhances the model’s generalization ability but also improves its performance in verification tasks.

The definition of the soft target cross-entropy loss function is as follows: assuming a batch of m samples, where all samples are represented as x, denoted as $x={\left\{\;x_{1,},\;x_{2},\;\ldots \;x_{m}\right\}}^{T}$, where $x_{i}$ represents the feature vector of the *i*-th sample. The feature vector of a sample has a dimension of n, and $x_{i}$ can be expressed as {$x_{i}^{\left(1\right)}$,$x_{i}^{\left(2\right)},\ldots x_{i}^{\left(n\right)}$}. Transforming $x_{i}$ input features into a normalized probability distribution is represented as ${\hat{y}}_{i}=softmax\left(x_{i}\right)$, where the calculation formula for each feature in ${\hat{y}}_{i}$ is as follows:

(4) $${\hat{y}}_{i}^{\left(j\right)}=\frac{e^{x_{i}^{\left(j\right)}}}{\sum_{k=0}^{n}e^{x_{i}^{\left(k\right)}}}\;\mathrm{for}\;j\;=\;1,2,\ldots n$$

A batch of m ${\hat{y}}_{i}$ samples forms $\hat{y}$, which can be represented as ${\left\{\;{\hat{y}}_{1},\;{\hat{y}}_{2},\;\ldots \;{\hat{y}}_{m}\right\}}^{T}$. As mentioned earlier, $y_{k}$ is obtained after applying Mixup for data augmentation, and a batch of m samples of $y_{k}$ forms y, represented as $\;{\left\{\;y_{1,},\;y_{2},\;\ldots \;y_{m}\right\}}^{T}$. Both y and $\hat{y}$ have a dimension of m×n. We take the logarithm of $\hat{y}$ and element-wise multiplication with y, then perform summation over rows and columns. Finally, we divide it by m to obtain the soft target cross-entropy loss for that batch. The calculation formula is as follows:

(5) $$l_{s}=\frac{-\sum_{i=1}^{m}\sum_{j=1}^{n}y⊙\log\hat{y}}{m}$$

The formula for calculating the center loss function is as follows:

(6) $$l_{c}=\frac{1}{2}{\sum_{i=1}^{m}\left\|x_{i}-c_{yi}\right\|}_{2}^{2}$$

Here, $c_{yi}$ represents the center of the category to which $y_{i}$ belongs. However, calculating $c_{yi}$ requires iteration through all samples in the training set to determine the corresponding category center. This means that during training, updating the centers of all classes for every batch is necessary. However, such an operation is impractical due to its computational complexity and time cost. In order to solve the above problems, the centers of all categories are not updated during mini-batch training. Instead, only the category centers that are related to the categories encountered in that batch are updated. Furthermore, to mitigate significant disturbances caused by a small number of mislabeled samples, a scalar α can be used to adjust the learning rate of the category center $c_{yi}$.

Based on the soft target cross-entropy function, the formula after adding a certain proportion of the center loss function is as follows:

(7) $$l=l_{s}+\lambda l_{c}=\frac{-\sum_{i=1}^{m}\sum_{j=1}^{n}y⊙\log\hat{y}}{m}+\frac{\lambda }{2}{\sum_{i=1}^{m}\left\|x_{i}-c_{yi}\right\|}_{2}^{2}$$

## 4. Experiment and Results

### 4.1. Dataset Description

The experiment utilizes two publicly available finger vein datasets: SDUMLA-HMT [41] from Shandong University (SDU dataset in short) and FV-USM [42] from Universiti Teknologi Malaysia (USM dataset in short). These two datasets serve as the basis for testing and validating the performance of the models and optimization algorithms utilized in this study.

#### 4.1.1. SDUMLA-HMT Database

The SDUMLA-HMT vein database, collected by Shandong University, comprises finger vein images from 106 individuals. Each participant provides vein images of the index, middle, and ring fingers from both the left and right hands, resulting in a total of 636 samples. The original size of each image is 320 × 240 pixels. We preprocess these images, including extracting the effective area and enhancing the image, resulting in an enhanced image size of 256 × 256. In the experiment, 80% of the data is allocated for model training, while the remaining 20% is used for testing.

#### 4.1.2. FV-USM Database

The FV-USM vein database, collected by Universiti Teknologi Malaysia, comprises finger vein images from 123 individuals (83 males and 40 females) aged between 20 and 52 years. Each participant provides vein images with a size of 320 × 240 pixels for the left-hand index finger, left-hand middle finger, right-hand index finger, and right-hand middle finger, resulting in a total of 492 samples. We process these images in a similar manner as per the image processing techniques used in the SDUMLA-HMT dataset. In the experiments, 80% of the data is allocated for training, while the remaining 20% is used for testing.

### 4.2. Training Details

The deep learning model used in this paper is implemented using the PyTorch deep learning framework. The experimental computer configuration and hardware environment are as follows: the CPU used is an AMD Ryzen 9 5900HX (The AMD Ryzen 9 5900HX is manufactured by Advanced Micro Devices, Inc. (AMD). AMD’s headquarters are located in Santa Clara, CA, USA. However, the silicon chips for the AMD Ryzen 9 5900HX are not made in-house by AMD but are manufactured by Taiwan Semiconductor Manufacturing Company (TSMC) in Taiwan), the GPU is an Nvidia RTX 3080 with 16 GB of video memory, and the operating system is Windows 10. In the training process, the Adam optimizer is employed to train the neural network model. To prevent the neural network from becoming trapped in local optima, the cosine annealing learning rate scheduler is employed. Initially, a relatively high learning rate of 0.0001 is used to facilitate rapid convergence, and as the number of iterations increases, the neural network gradually converges. The learning rate is cosine-annealed down to 1 × 10^−6^. Given that achieving the global optimum using the cosine annealing algorithm requires a significant number of iterations, the experiment is set to run for 3000 iterations. Model parameters are initialized using a zero-mean Gaussian distribution with a standard deviation of 0.01.

Accuracy (Acc), False Acceptance Rate (*FAR*), False Rejection Rate (*FRR*), and Equal Error Rate (EER) [43] are used to assess the performance of the finger vein recognition system. *FAR* is determined by comparing the similarity between the feature vectors of different fingers. If the similarity exceeds a predefined threshold, it is considered a false acceptance. The *FAR* is calculated using the Formula (8), where the number of false acceptances is denoted as $N_{A}$ and the total number of comparisons is *N*. The *FRR* involves comparing the similarity between feature vectors of different samples from the same finger. If the similarity falls below a predefined threshold, it is considered a false rejection. The *FRR* is calculated using the Formula (9), where the number of false rejections is also denoted as $N_{R}$, and the total number of comparisons remains *N*. The similarity threshold varies from 0 to 1, resulting in corresponding changes in the *FAR* and *FRR* curves. When *FAR* equals *FRR*, the Equal Error Rate (EER) is obtained.

(8) $$FAR=N_{A}/N\cdot 100\%$$

(9) $$FRR=N_{R}/N\cdot 100\%$$

### 4.3. Results Evaluation and Comparison

#### 4.3.1. Parameter Confirmation Experiment

To improve model recognition accuracy, we employ a combination of soft target cross-entropy loss and center loss as the model’s loss function. The proportion λ of the center loss function and the learning rate α need to be determined through experiments. We employ a grid search approach to determine the optimal parameters. In the experiment, we initially set a fixed value for the proportion λ and then changed the learning rate α for training. The learning rate α primarily affects the convergence speed and stability of the model. We start with a relatively large value of 0.001 for the first α and successively decrease it until reaching 0.00001. Ideally, as α decreases, the convergence speed slows down, but the accuracy shows some improvement. Subsequently, we adjust the parameter λ, which mainly balances the trade-off between center loss and soft target cross-entropy loss. A larger λ indicates a greater emphasis on intra-class compactness, while a smaller λ emphasizes inter-class separability. In the experiment, we systematically reduced λ, gradually increasing inter-class separability. Therefore, ideally, the accuracy continues to increase until a certain point where further reduction in λ does not result in accuracy improvement. The accuracies present in Table 1 and Table 2 are the results of experiments conducted on two different datasets using different values of α and λ. From the tables, it can be observed that when λ is set to 0.0001, along with a continuous decrease in α, the accuracy no longer increases when α is reduced to 0.00001, indicating the optimal experimental result is achieved. When λ is further reduced to 0.00001, regardless of how much α decreases, the accuracy remains unchanged, indicating the optimal experimental result.

#### 4.3.2. Improved Component Experiment

In this section, a series of experiments is conducted to examine the impact of various improved components in our finger vein recognition algorithms on recognition rates and equal error rates. These results contribute to evaluating the effectiveness of these improvements and provide insights for further optimization efforts. The first experiment employs unprocessed original images for feature extraction and matching using MobileViT, with SGD as the optimizer and a fixed learning rate. This experiment is referred to as the “Original Image”. The second experiment, labeled as “Dynamic LR”, involves using Adam as the optimizer and employs cosine annealing learning rate scheduling. The third experiment involves extracting features from the Mini-ROI region of the original images and is denoted as “Mini-ROI”. The fourth experiment evaluates the results obtained after training with a lightweight model introduced in this paper, utilizing the soft target cross-entropy loss function. This experiment is abbreviated as “Lightweight”. The fifth experiment, referred to as “Center loss”, incorporates a certain proportion of the center loss function on top of the soft target cross-entropy loss function. The testing of each component is conducted on the foundation of all preceding components.

**Figure 9:**
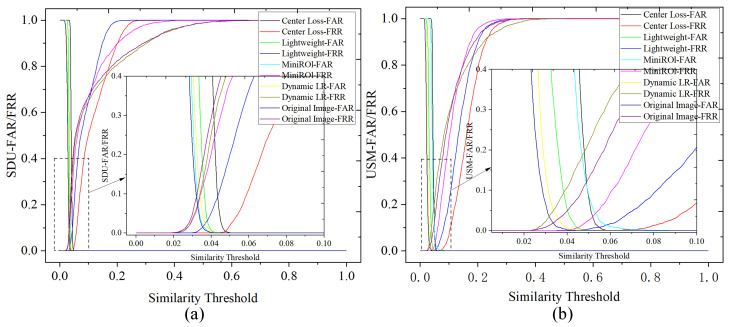
(**a**) SUD-FAR/FRR curve; (**b**) USM-FAR/FRR curve.

Figure 9 illustrates the *FAR* and *FRR* curves of different experiments on the two datasets against the different similarity threshold values used. The EER of an experiment is observed as the coordinate of where the *FAR* and *FRR* curves intersect. A lower EER indicates better performance. From the graph, it can be observed that with the gradual addition of each improvement component, the vertical axis of the intersection point of FAR and FRR decreases, resulting in a smaller EER. The optimal result is achieved when the improvement components include the central loss function.

**Figure 10:**
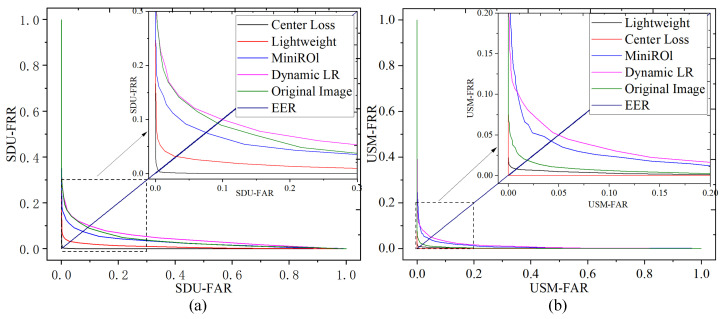
(**a**) SDU DET Curve; (**b**) USM DET Curve.

Figure 10 plots the *FRR* curve of different experiments on the two datasets against their *FAR*. The resulting curve is known as the Detection Error Tradeoff (DET) curve. The performance evaluation method for the DET curve is as follows: the smaller the area enclosed by the DET curve and the horizontal axis, the better the overall algorithm performance. From the graph, it can be observed that as various improvement components are gradually added, the area enclosed by the DET curve becomes smaller. When the improvement components include the central loss function, the area becomes the smallest, indicating the best performance.

Table 3 shows the test results after incorporating various improvement components. It is evident that dynamic learning rate adjustment offers certain advantages over fixed learning rates, with an increase of 0.4% and 0.2% in recognition accuracy on the SDU and USM datasets, respectively. Images with Mini-ROI feature extraction exhibit significant advantages over the original images, enhancing recognition accuracy by 3.5% and 0.2% on the SDU and USM datasets, respectively, while reducing EER by 2.31% and 0.58%. Due to the FV-MViT model’s ability to extract more local feature information and perform global information extraction after local feature extraction, FV-MViT outperforms MViT by 1.1% and 0.4% in recognition accuracy on the SDU and USM datasets, respectively, while reducing EER by 4.63% and 4.11%. As the soft target center cross-entropy loss function can facilitate clustering, it enhances recognition accuracy by 0.6% and 0.2% on the SDU and USM datasets, respectively, compared to the soft target cross-entropy loss function, while reducing EER by 2.54% and 0.14%, respectively.

#### 4.3.3. Comparison against Mainstream Transformer-Related Frameworks

Table 4 displays the results of the proposed method against mainstream transformer-related frameworks applied to finger vein recognition, where all experimental outcomes are conducted under identical experimental conditions. Since Vision Transformer extracts global features, the accuracy increases on larger datasets and decreases on smaller ones, resulting in the lowest accuracy when applied to finger vein datasets. Swin Transformer, which builds upon ViT by utilizing movable windows to extract local features, also achieves significant performance on these datasets. MobileViT, a fusion of CNN and Transformer, effectively enhances finger vein recognition accuracy. The proposed FV-MViT, built upon MobileViT, extracts more features and boosts vein recognition accuracy. In comparison, FV-MViT shows an improvement of 1.42% and 0.61% in recognition accuracy compared with MobileViT on the SDU and USM datasets, respectively, while also reducing EER by 5.37% and 4.25%, respectively. Regarding the number of parameters, FV-MViT showcases considerable advancement compared with both ViT and Swin Transformer. Despite having a higher parameter count than MobileViT, FV-MViT achieves a notable improvement in recognition rate. Among all the compared methods, MobileNetV2 has the least number of parameters and FLOPS, with the lowest latency, but fails to achieve a recognition accuracy of 99%.

#### 4.3.4. Comparison of Experimental Results for Various Vein Recognition Networks

Table 5 presents a comparative analysis of the algorithm proposed in this paper against recent deep learning-based vein recognition algorithms. The first two studies utilize Convolutional Neural Networks (CNNs), achieving high recognition accuracy. Huang et al. [32] employed a Transformer as the primary feature extractor, while Li et al. [44] used a Vision Transformer for feature extraction. From the table, it is evident that our lightweight vein recognition algorithm, which combines CNN and Transformer, outperforms algorithms using Transformer as the primary feature extractor. In comparison with Li [44], our approach achieves a substantial increase in recognition accuracy. Specifically, on the SDU and USM datasets, our algorithm improves recognition accuracy by 7.29% and 0.27%, respectively, while also reducing the EER by 0.55% and 0.096%. Notably, our approach achieves competitive results in terms of Params and Latency in Table 5, even surpassing the state-of-the-art (SOTA) methods.

#### 4.3.5. Comparison of Experimental Results for Various Loss Functions

Table 6 provides a comparative analysis of the proposed loss functions in this paper against other loss functions. A noticeable enhancement in recognition rates is evident in the table, attributed to the superior generalization of the soft target cross-entropy loss function over the original SoftMax loss function. Specifically, the performance of soft target cross-entropy outperforms SoftMax on the SDU and USM datasets, augmenting the accuracy by 0.72% and 0.81%, respectively, and reducing the EER by 10.38% and 5.38%, respectively. The center loss function, designed to diminish intra-class distances and augment inter-class distances, yields substantial performance improvements when combined with both the SoftMax and soft target cross-entropy loss functions. Coupled with SoftMax, center loss enhances accuracy by 0.88% on the SDU dataset and 0.81% on the USM dataset, concurrently reducing the EER by 11.01% and 4.03%, respectively. When integrated with the soft target cross-entropy loss function, center loss elevates accuracy by 0.64% on the SDU dataset and 0.21% on the USM dataset, while also decreasing the EER by 2.54% and 0.14%, respectively. These results underscore the significant impact of the chosen loss function on recognition accuracy and EER, with center loss demonstrating strong potential for improving vein recognition performance. The combination of center loss and soft target cross-entropy loss emerges as the most effective approach, delivering the optimal performance.

## 5. Conclusions

We present a novel FV-MViT model that, compared with the original MobileViT, demonstrates improved recognition accuracy by 1.42% and 0.61% on the SDU and USM datasets, respectively. Additionally, it reduces the equal error rate by 5.37% and 4.25%, respectively, with a parameter size of 5.26 M and a reduction in delay by 1 ms. Concurrently, extensive experiments conducted on two finger vein datasets demonstrate that the proposed FV-MViT exhibits competitive performance compared to the state-of-the-art (SOTA). The FV-MViT not only effectively enhances the accuracy of finger vein recognition but also aligns with the real-time processing and low-cost requirements for deployment on embedded systems. It offers advantages such as high accuracy, lightweight portability, and low latency, making it valuable for practical applications. These benefits facilitate easy deployment in mobile products, contribute to cost reduction, and encourage the widespread adoption and application of finger vein recognition on a large scale.

Despite the promising results achieved by the proposed Mini-ROI extraction technique, the FV-MViT architecture, and the soft target center cross-entropy loss function, there is potential for additional improvements through ongoing research efforts. In future work, the following aspects remain worth further investigation: (1) Optimizing the parameter counts of FV-MViT to potentially be lower than that of MobileNetV2. (2) The feature extraction time of FV-MViT is 10 ms, and there is further room for optimization in the feature extraction time.

## Figures and Tables

**Table 1:** SDU-Acc experimental results with different parameters α and λ.

	λ	0.01	0.001	0.0001	0.00001
α					
0.001		98.74%	99.21%	99.37%	99.53%
0.0001		99.05%	99.21%	99.37%	99.53%
0.00001		99.05%	99.21%	99.53%	99.53%
0.000001		99.05%	99.37%	99.53%	99.53%

**Table 2:** USM-Acc experimental results with different parameters α and λ.

	λ	0.01	0.001	0.0001	0.00001
α					
0.001		98.17%	98.98%	99.79%	100.00%
0.0001		98.37%	99.39%	99.79%	100.00%
0.00001		98.37%	99.79%	100.00%	100.00%
0.000001		98.98%	99.79%	100.00%	100.00%

**Table 3:** Recognition results of our method on the finger vein databases.

Components	SDU-Acc	SDU-EER	USM-Acc	USM-EER
Original Image	93.87%	9.37%	98.98%	1.80%
Dynamic LR	94.33%	9.95%	99.19%	4.85%
Mini-ROI	97.80%	7.64%	99.39%	4.27%
Lightweight	98.89%	3.01%	99.79%	0.16%
Center Loss	99.53%	0.47%	100.00%	0.02%

**Table 4:** Results of different mainstream networks on SDU and USM datasets.

Method	SDU-Acc	SDU-EER	USM-Acc	USM-EER	#Params.	FLOPS	Latency
VisionTransformer [17]	83.96%	9.30%	87.40%	4.51%	37.98 M	4.75 G	6.99 ms
SwinTransformer [18]	93.55%	6.62%	98.58%	2.86%	63.49 M	15.03 G	24.00 ms
MobileNetV2 [26]	96.10%	4.35%	98.98%	3.96%	1.42 M	0.06 G	6.00 ms
MobileViT [19]	98.11%	5.84%	99.39%	4.27%	5.09 M	1.82 G	11.00 ms
FV-MViT	99.53%	0.47%	100.00%	0.02%	5.26 M	1.84 G	10.00 ms

**#Params.** represent the parameter count in the model.

**Table 5:** Recognition results of our proposed method and other algorithms.

Method	SDU-Acc	SDU-EER	USM-Acc	USM-EER	#Params.	FLOPS	Latency
Huang et al. [30] (2021)	98.61%	1.18%	99.77%	0.49%	-	-	-
Shen et al. [27] (2022)	99.3%	1.13%	-	-	-	-	14.2 ms
Huang et al. [32] (2022)	89.29%	3.25%	99.59%	0.54%	50 M	1.7 G	-
Li et al. [44] (2023)	92.21%	1.022%	99.73%	0.116%	-	-	-
Ours	99.53%	0.47%	100.00%	0.02%	5.26 M	1.84 G	10.0 ms

**#Params.** represent the parameter count in the model.

**Table 6:** Performance of different loss functions on SDU and USM datasets.

Method	SDU-Acc	SDU-EER	USM-Acc	USM-EER
SoftMax	98.17%	13.39%	98.98%	5.54%
SoftMax + center loss	99.05%	2.38%	99.79%	1.51%
Soft target cross-entropy	98.89%	3.01%	99.79%	0.16%
Soft target cross-entropy + center loss	99.53%	0.47%	100.00%	0.02%

## Data Availability

The data presented in this study are publicly available in the SDUMLA-HMT and FV-USM, reference number [41,42].
